# Advances in the structural characterization and pharmacological activity of Salvia miltiorrhiza polysaccharides

**DOI:** 10.3389/fchem.2025.1492533

**Published:** 2025-03-14

**Authors:** Ke Yang, Yi-Jun Liu, Jia-Ning Zhang, Ya-Jing Chen, Jian Yang, Jun-Ping Xiao, Han-Bin Lin, Hong-Jun Yang

**Affiliations:** ^1^ Zhejiang Provincial Key Laboratory of Biometrology and Inspection and Quarantine, College of Life Science, China Jiliang University, Hangzhou, China; ^2^ Beijing Key Laboratory of Traditional Chinese Medicine Basic Research on Prevention and Treatment for Major Diseases, Experimental Research Center, China Academy of Chinese Medical Sciences, Beijing, China; ^3^ State Key Laboratory Breeding Base of Dao-di Herbs, National Resource Center for Chinese Materia Medica, China Academy of Chinese Medical Sciences, Beijing, China; ^4^ Dexing Research and Training Center of Chinese Medical Sciences, Dexing, China; ^5^ Jiangxi Prozin Pharmaceutical Co., Ltd., Ji’an City, Jiangxi, China; ^6^ Zhongshan Institute for Drug Discovery, Zhongke Zhongshan Pharmaceutical Innovation Research Institute (SIMM CAS), Zhongshan, Guangdong, China

**Keywords:** *Salvia miltiorrhiza* polysaccharide, extraction, purification, structural characteristics, pharmacological activity

## Abstract

**Background:**

*Salvia miltiorrhiza* Bunge is the dried root and rhizome of *Salvia miltiorrhiza* Bunge, a labiatae plant. *Salvia miltiorrhiza* polysaccharide (SMP) is the main active component of *Salvia miltiorrhiza* Bunge. The extraction methods of SMP mainly include water extraction, ultrasonic extraction, enzyme extraction, microwave-assisted extraction and acid-base extraction. It is mainly composed of glucose, arabinose, rhamnose, galactose and other monosaccharides. SMP has a variety of biological activities, including immune regulation, anti-tum, anti-oxidation, myocardial protection, liver protection and so on.

**Purpose:**

*Salvia miltiorrhiza* polysaccharide is widely used in nutraceuticals and pharmaceuticals, and has high research value. Natural polysaccharides are non-toxic, soluble in water, and have a wide range of biological activities, so they have broad research prospects.

**Methods:**

The data was collected using different online resources including PubMed, Google Scholar, and Web of Science using keywords given below.

**Results:**

In the past decades, various reports have shown that the pharmacological activities of *Salvia miltiorrhiza* polysaccharides have good effects, and the side effects are small.

**Conclusion:**

This paper summarizes the extraction and purification methods, molecular weight, monosaccharide composition, glycosidic linkage, pharmacological activity, toxicity, product development, clinical research and other contents of *Salvia miltiorrhiza* polysaccharides in recent years, providing a theoretical basis for further study of *Salvia miltiorrhiza* polysaccharides.

## 1 Introduction

**FIGURE 1:**
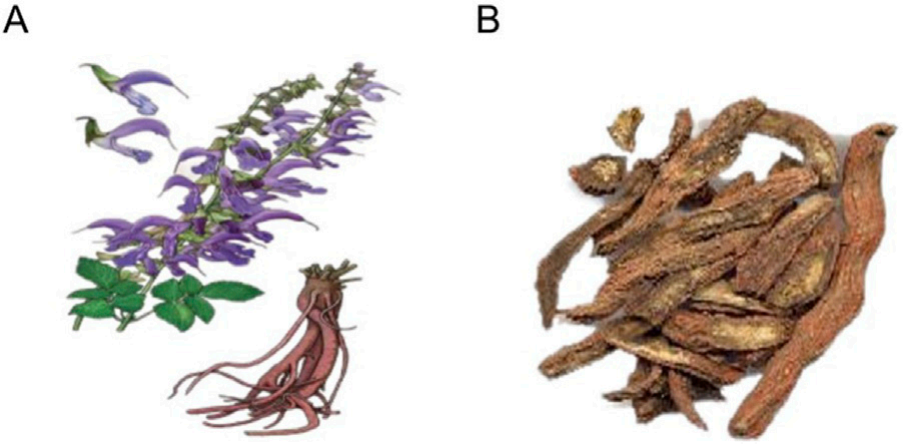
**(A)** Original medicinal materials and **(B)** decoction pieces of SM.

*Salvia miltiorrhiza* is the dried root and rhizome of *Salvia miltiorrhiza* Bunge, which belongs to the Labiatae family. It is recorded in the Chinese Pharmacopoeia 2020, which stipulates that the dried root and rhizome of *Salvia miltiorrhiza* Bunge are the only source (Nai et al., 2021). The plant body is brownish red or dark brownish red, with longitudinal wrinkles. The decoction sheet is round or oval thick, the phloem is brownish red, and the xylem is grayish yellow or purplish brown, with yellowish white radial texture (Figure 1). It is mainly produced in Sichuan, Hubei, Henan, Shandong, Shaanxi and other Chinese provinces (Guo et al., 2002). *Salvia miltiorrhiza* was first recorded in the Shennong Classic of Materia Medica, and was listed as the top grade of medicinal material. It was officially included in the Chinese Pharmacopoeia in 1963 and has been used in clinical trials for more than 2,000 years (Guo et al., 2014). It has the effect of activating blood circulation, removing blood stasis and relieving pain, clearing the heart and removing annoyance, cooling blood and eliminating carbuncle. It is often used as a drug for promoting blood circulation and removing blood stasis in clinic (Jia et al., 2019). Modern pharmacological research shows that *Salvia miltiorrhiza* can treat various diseases, including diabetes (Yin et al., 2021), cardiovascular disease (Wang et al., 2017), osteoporosis (Guo et al., 2014), diabetic nephropathy (Xie et al., 2021), etc.

To date, over 100 chemical components have been isolated from *Salvia miltiorrhiza*, including tanshinone, salvianolic acid, tanshinol, polysaccharide, and others (Wang et al., 2017). Many studies have shown that *Salvia miltiorrhiza* polysaccharide is one of the main components of *Salvia miltiorrhiza* (Yongming and Phamacy, 2016). It exhibits a wide range of biological activities, such as anti-tumor (Liu et al., 2013), anti-oxidation (Jiang et al., 2014), anti-virus (Chen et al., 2017), immune regulation (Wang et al., 2014), hypoglycemic and hypolipidemic (Geng et al., 2015a). Consequently, it finds extensive application in health products and pharmaceuticals, underscoring its significant research value. Natural polysaccharides are characterized by their non-toxicity, water solubility, and broad spectrum of biological activities, thus offering promising research prospects (Zhang et al., 2018). This review summarizes the progress in the extraction, purification, chemical composition, structure and pharmacological action of *Salvia miltiorrhiza* polysaccharides. And it provides a reference for further study on the efficacy relationship of *Salvia miltiorrhiza* polysaccharides and the development and utilization of *Salvia miltiorrhiza* polysaccharides.

## 2 Extraction of *Salvia miltiorrhiza* polysaccharide

Polysaccharides are a kind of macromolecular metabolites containing many hydroxyl groups, which are polymerized from many small monosaccharides. Polysaccharides can form hydrogen bonds with water molecules, so they can be soluble in water, but not in organic solvents. There are many extraction methods for polysaccharides, and different extraction methods can be selected according to the structural properties of polysaccharides. The common extraction methods of polysaccharides include water extraction (Zeng et al., 2019a), ultrasonic extraction (Maran and Priya, 2014), enzyme extraction (Zhang et al., 2016), microwave-assisted extraction (Al-Dhabi and Ponmurugan, 2020) and acid-base extraction (Han and Wang, 2013). Different extraction methods have their own advantages and disadvantages. The extraction method of *Salvia miltiorrhiza* polysaccharides is similar to that of other polysaccharides, including solvent extraction, ultrasonic extraction, enzyme extraction and microwave assisted extraction.

### 2.1 Water extraction method

The water extraction method has the advantages of operability, pollution-free, simple method, low cost, etc. It is widely used for the extraction of polysaccharides and is suitable for industrial application. But at the same time, it requires high extraction temperature, takes a long time, has low efficiency, low safety, and is difficult to purify, while repeated continuous extraction is very easy to damage the chemical structure of polysaccharides and affect the stability of polysaccharides (Yuan, 2015).

Jiang et al. used Box Behnken design to optimize the extraction process of polysaccharides from *Salvia miltiorrhiza* residue. The results showed that the extraction time, extraction temperature and water to material ratio were the important factors affecting the extraction rate of polysaccharides. The optimized extraction condition was as follows: the extraction time was 2.6 h, the extraction temperature was 89°C, the volume ratio of water to raw material was 32:1, and the extraction rate of crude polysaccharide was around 27.32% (Jiang et al., 2015). Cai’s research showed that the optimal extraction process of *Salvia miltiorrhiza* polysaccharide is soaking in water for 2 h, heating to boiling for 30 min, filtering, then decocting for 25 min and filtering, collecting the filtrate twice, the concentrated solution contains 2 g/mL of *Salvia miltiorrhiza*, adding anhydrous ethanol to make its concentration up to 80%, placed at a constant temperature of 20°C for 4 h, and centrifuged to precipitate to obtain *Salvia miltiorrhiza* polysaccharide (Cai et al., 2010).

### 2.2 Ultrasonic extraction

Ultrasound technology can induce deformation and rupture of tissues and facilitating the release of intracellular contents, thus promoting the dissolution of active components in cells. This process is characterized by its rapidity, precision, and stability, which is conducive to the dissolution of effective components. The method requires no heating and has a high extraction rate. But high power will destroy the polysaccharide structure, extracellular substances cause separation difficulties (Huang, 2010).

Jiang et al. optimized the extraction process of polysaccharides from *Salvia miltiorrhiza* using response surface methodology. The optimized extraction process was the extraction temperature of 54°C, the ultrasonic power of 180 W, the extraction time of 32 min, and the extraction rate of up to 40.54% (Jiang et al., 2014). Zhao optimized the ultrasonic extraction process of *Salvia miltiorrhiza* polysaccharide through orthogonal test. Among them, the ratio of material to liquid has the greatest impact on the extraction rate of *Salvia miltiorrhiza* polysaccharide. The optimal extraction process conditions was: the ratio of material to liquid (m/V) was 1∶12, the extraction temperature was 50°C, the extraction time was 40 min, and the extraction times were 3. Under this condition, the extraction rate of *Salvia miltiorrhiza* polysaccharide was 5.43% (Zhao, 2014).

### 2.3 Enzymatic extraction

Enzymatic extraction has the advantages of convenience, specificity, easy removal of impurities, high efficiency, cost saving and energy consumption reduction. Based on this, enzymatic extraction has broad application space. But the cost of enzyme extraction is very high (Zhang et al., 2010). Depending on the specificity of the enzyme, the complex enzyme is used in the experiment to coordinate the relationship between substrate, inhibitor and enzyme concentration, according to its required pH, temperature and time.

Cai et al. optimized the extraction process of *Salvia miltiorrhiza* polysaccharide by cellulase method. The results showed that the optimal extraction process of *Salvia miltiorrhiza* polysaccharide by cellulase method was temperature 60°C, enzyme amount 5%, and extraction time 120 min. Under this condition, the extraction rate of polysaccharide was 108.9 g/kg (Cai et al., 2008). Yang et al. optimized the extraction process of polysaccharides from *Salvia miltiorrhiza* by cellulase using response surface methodology. On the basis of single factor investigation and response surface methodology, they finally concluded that the optimal enzyme extraction process was 0.5% enzyme addition, 65°C enzymatic hydrolysis temperature, 120 min extraction time, and the extraction rate of polysaccharide was 2.59 mg/g (Yang and Zhang, 2016).

### 2.4 Microwave assisted method

Microwave energy penetrates the cell wall and reaches the cell interior in the presence of a solvent. The elevated temperature and pressure facilitate the absorption of microwave energy by both the solvent and the intracellular components. When the internal pressure exceeds the structural integrity of the cell, the cell wall ruptures and the cell material flows out and dissolves in the solvent. So as to improve the extraction rate. Microwave extraction has the advantages of simplifying operation steps, saving solvent, safety and pollution-free, and improving extraction rate. However, the disadvantage of this method is consumption, and the polysaccharide structure is easy to be destroyed (Jun-Mei et al., 2010).

According to Meng et al., the microwave-assisted extraction process of crude polysaccharides from *Salvia miltiorrhiza* (SMPs) was optimized by investigating four independent variables, microwave power, extraction time, solvent solid ratio and ethanol concentration. The results showed that the optimum extraction conditions were: microwave power 1200W, extraction time 12 min, solvent to solid ratio 38%, ethanol concentration 86%, and the final extraction rate of crude polysaccharide 14.11% (Meng et al., 2022). Zhao also optimized the extraction of polysaccharides from *Salvia miltiorrhiza* by microwave through single factor experiment and orthogonal design. By taking the extraction yield of polysaccharides as the evaluation index, the material-to-liquid ratio, extraction temperature, extraction time, and number of extractions were optimized. The optimum extraction conditions were as follows: 1: 12 g/mL, 50°C, 40 min, achieving a polysaccharide extraction yield of 5.43% (Zhao, 2014) (Table 1).

**TABLE 1:** The advantage and disadvantage of extraction of *Salvia miltiorrhiza* polysaccharide.

Extraction method	Advantage	Disadvantage
Water extraction method (Yuan, 2015)	Operability, pollution-free, simple method, low cost, etc.	High extraction temperature, long time, low efficiency, low safety, difficult purification, the chemical structure of polysaccharide is easy to destroy, and low stability of polysaccharide
Ultrasonic extraction (Huang, 2010)	Fast, accurate, stable, high extraction rate and conducive to dissolution of active ingredients	Destroy the structure of polysaccharide and make extracellular substances difficult to separate
Enzymatic extraction (Zhang et al., 2010)	Convenient, specific, easy to remove impurities, high efficiency, cost saving, low energy consumption	High cost
Microwave assisted method (Jun-Mei et al., 2010)	Simple operation procedure, saving solvent, safe and pollution-free, and improving the extraction rate	Large consumption, and the structure of polysaccharide is easily destroyed

## 3 Purification of *Salvia miltiorrhiza* polysaccharide

The extraction of *Salvia miltiorrhiza* was conducted. Crude polysaccharide generally contains protein, pigment and other impurities. The polysaccharide component can be separated after further purification through protein removal, pigment removal and other impurity removal processes. Common methods for protein removal include Sevag method (Yang et al., 2022), CaCl_2_ method (Cheng and Huang, 2018), NaCl method (Huang et al., 2011), trichloroacetic acid method (Chen et al., 2018), enzymolysis method (Zeng et al., 2019b), etc. The separation and purification of polysaccharides are often carried out by chromatography (Lv et al., 2020), ethanol precipitation (Wang et al., 2018b), ultrafiltration (Eder et al., 2021) and other methods. Chromatography is the most widely used method to classify and purify polysaccharides. It is divided into ion exchange chromatography (Ren and Liu, 2020) and gel filtration chromatography (Pawlaczyk-Graja et al., 2019).

**FIGURE 2:**
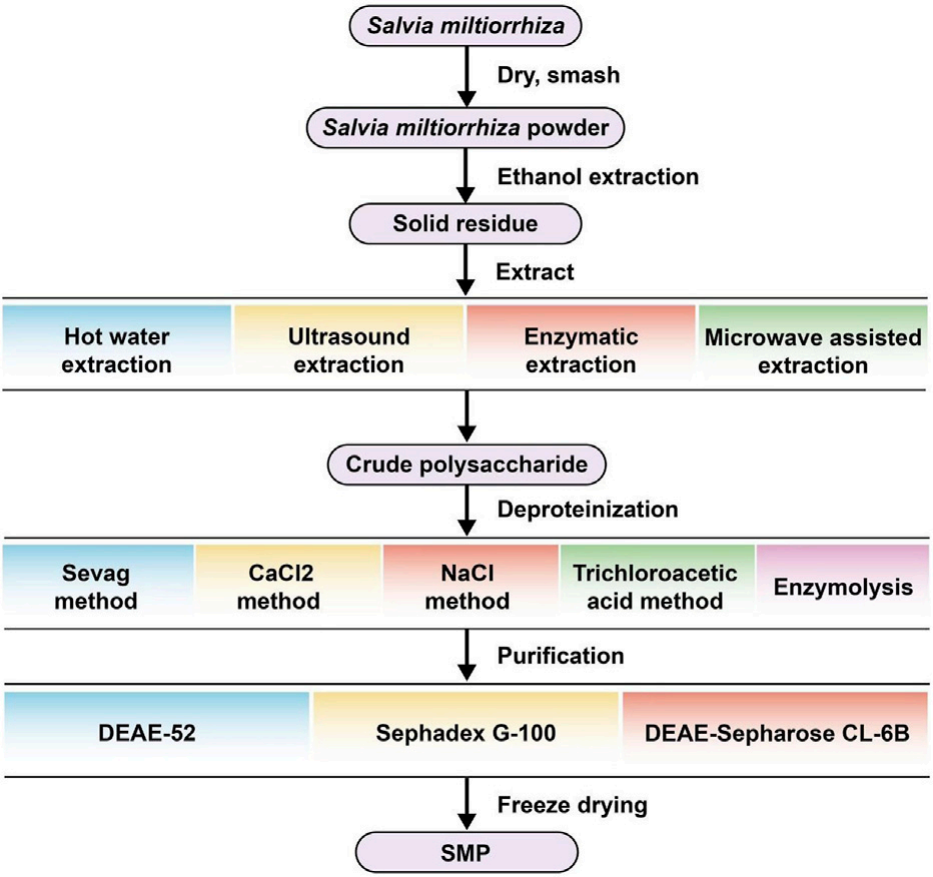
Extraction, separation and purification process of *Salvia miltiorrhiza* polysaccharide.

*Salvia miltiorrhiza* polysaccharides can be separated using DEAE Sepharose CL-6B, DEAE-52, Sephadex G-100, and similar materials. Jiang et al. extracted crude polysaccharides from *Salvia miltiorrhiza* using hot water, removed proteins with papain, filtered the solution through a 0.45 μm filter, and purified it via DEAE Sepharose CL-6B column chromatography. Further purification was performed using Sephadex G-100 gel permeation chromatography, resulting in the isolation of Salvia miltiorrhiza polysaccharide SMWP-1 (Jiang et al., 2015). Jiang et al. also used 0.45 μM membrane filtration and purification after extracting crude polysaccharide and removing protein. Then they wash with 0.5 mol/L NaCl solution at a flow rate of 2.5 mL/min, collect the washing liquid fraction, concentrate, dialysis, freeze dry, and further purify it by size resistance chromatography (deionized water is used as the washing liquid) penetrated into the chromatographic column through Sephadex G-100 gel to obtain the polysaccharide fraction SMWP-U&E (Jiang et al., 2020). Tang et al. obtained the crude polysaccharide of *Salvia miltiorrhiza* by water extraction and ethanol precipitation, subsequently purified them using macroporous resin and ion exchange chromatography. Crude polysaccharide prepared by AB-8 column was further separated and refined by DEAE-52 ion exchange chromatography to obtain SMP1, SMP2 and SMP3 (Wei et al., 2010). In conclusion, *Salvia miltiorrhiza* polysaccharide obtained by the above purification method can be used for chemical composition research and structural analysis. The extraction, separation and purification flow chart of *Salvia miltiorrhiza* polysaccharide is shown in Table 2; Figure 2.

**TABLE 2:** Extraction, purification method, yield, molecular weight and monosaccharide composition of *Salvia miltiorrhiza* polysaccharide.

Fraction name	Extraction method	Purification method	Yield%	Molecular weight(Da)	Monosaccharide composition
SMWP-U&E (Jiang et al., 2020)	Water extraction	DEAE Sepharose CL-6B, Sephadex G-100, T-1000, T-500, T-200, T-100 and T-50		5.07 × 10^5^	Ara:Fru:Man:Glc:Gal = 3.72:4.11:6.18:32.08:53.91
SMPA (Wang et al., 2014)	Hot water extraction 3 times, 95% ethanol precipitation, Protein removal by sevag method	DEAE-52 cellulose column and Sephadex G-100 gel filtration column	5.23	4.3 × 10^5^	Glc∶Ara∶Xyl∶Man∶GalUA = 1.42∶2.14∶1.16∶2.1∶1
SMP (Chen et al., 2017)	Water extraction 3 times, ethanol precipitation, Sevag reagent treatment		1.8		Ara:Gal:Glu:Rham:GalUA = 4.79:8.24:3.26:1:6.52
SMP1 (Meng et al., 2022)	Microwave assisted treatment	DEAE Sepharose Fast Flow and Sephadex G-100	14.11	6,087	Glu:Gal:Fru = 1:1.67:1.12
PSMP-2 (Jing et al., 2022)		DEAE-52 and Sephadex G-100		1.28 × 10^6^	Rha:GalA:Gal:Ara = 6.15:55.98:21.27:16.69
SMPs (Xiang et al., 2018)	Ultrasonic method		3.417		
SMPs (Yanhua et al., 2015)	Microwave assisted method, ethanol precipitation, Sevag reagent treatment		10.1161		
SMP-U1 (Jiang et al., 2014)	Ultrasonic extraction	Sephadex G-100	40.54	5.69 × 10^5^	Man: Rib: Xyl: Ara: Glu: Gal = 1.95:0.22:0.10:1.57:1.45:1.34
SMWP-1 (Jiang et al., 2015)	Hot water extraction	DEAE-Sepharose CL-6B, Sephadex G-100	27.32	5.27 × 10^5^	Glu:Xyl:Man:Gal = 0.34:0.28:0.27:0.11
SMP (Wang et al., 2018a)	Hot water extraction			1.2 × 10^5^	Gal:Glc:GalUA = 15.03:7.14:1.00
SMP-W1 (Liu et al., 2013)	Hot water extraction	DEAE, Sephadex G-100		6.9 × 10^5^	Man:Rha:Ara:Glu:Gal = 2.14:2.35:1.27:0.99:1.11
SMP (Chen et al., 2017)	Hot water extraction 3 times, ethanol precipitation at 95°C, protein removal by sevag		1.8		Glc∶Gal∶Ara∶Rha:GalUA = 3.26∶8.24∶4.79∶1∶6.52
SMPW1 (Zhang et al., 2012)	Hot water extraction 3 times	DEAE-Sephadex A-50, Sephadex G-200	6.9		
SMP 1 (Song et al., 2013)	Hot water extraction 3 times, protein removal by sevag method	DEAE cellulose column, Sepharose CL-6B gel column		5.5 × 10^5^	Glc∶Gal∶Ara∶Xyl∶Man∶Fuc = 1.2∶1.0∶1.3∶1.5∶1.9∶0.3
SMP (Xiao-Ni et al., 2016)	Hot water extraction		1.49		
SMP (Wang et al., 2007)	Hot water extraction	11.91–37.62			Glc∶Gal∶Ara∶Rha∶Xyl∶Man∶Rib = 12.7∶58.8∶15.3∶2.8∶1.0∶4.2∶8.5
SMPS (Yang and Zhang, 2016)	Enzyme extraction method		0.259		
SMPS (Wang et al., 2015)	Ultrasonic extraction		4.73		
SMPS (Gong, 2015)	Enzyme extraction method		13.36		
SMPS (Meng and Wang, 2009)	Ultrasonic extraction		8.72		
SMPS (Zhao, 2014)	Ultrasonic extraction 3 times		5.43		
SMPS (Cong-Ping et al., 2007)	Hot water extraction		2.503		
SMPS (Cai et al., 2008)	Enzyme extraction method		10.89		
SMPS (Wu et al., 2007)	Ultrasonic extraction		6.42		

## 4 Structural characteristics of *Salvia miltiorrhiza* polysaccharides

Various extraction, separation, and purification methods significantly influence the monosaccharide composition of *Salvia miltiorrhiza* polysaccharides. The structure of polysaccharides determines the properties of polysaccharides, so it is of great significance to study the structure of polysaccharides. The structure analysis of *Salvia miltiorrhiza* polysaccharide mainly includes its monosaccharide composition, molecular weight, glycosidic bond connection, etc.

### 4.1 Monosaccharide composition

Because the polysaccharide has no conjugated system and no ultraviolet absorption, the polysaccharide is usually hydrolyzed into monosaccharides before being determined by instrumental analysis technology when analyzing the monosaccharides composition of *Salvia miltiorrhiza* polysaccharide. High performance liquid chromatography (HPLC) (Wang et al., 2016), high-performance liquid chromatography-mass spectrometry (HPLC-MS) (Zhao et al., 2019), high-performance anion exchange chromatography (HPAEC-PAD) (Giannelli et al., 2020), high performance capillary electrophoresis HPCE (Ma et al., 2017), gas chromatography-mass spectrometry (GC-MS) (Grace et al., 2013) and other chromatographic methods are commonly used to determine monosaccharide composition. Meng et al. utilized microwave-assisted extraction followed by continuous purification using DEAE Sepharose Fast Flow and Sephadex G-100 chromatography to obtain SMP1 from Salvia miltiorrhiza polysaccharides, which comprised glucose, galactose, and fructose in a molar ratio of 1:1.67:1.12 (Meng et al., 2022). Wang et al. extracted the crude polysaccharide from *Salvia miltiorrhiza* by water extraction and ethanol precipitation, and separated and purified the polysaccharide components by DEAE-52 cellulose column and Sephadex G-100 gel filtration column. After hydrolysis by trifluoroacetic acid and derivatization of 1-phenyl-3-methyl-5-pyrazolone (PMP), The monosaccharide composition of the polysaccharide and its molar ratio were determined by HPLC method: glucose: arabinose: xylose: mannose: galacturonic acid = 1.42: 2.14: 1.16: 2.10: 1 (Wang et al., 2014). This indicates that *Salvia miltiorrhiza* polysaccharides primarily consist of monosaccharides such as glucose, arabinose, rhamnose, and galactose, with varying compositions and molar ratios depending on the extraction, separation, purification, and analytical methods employed.

### 4.2 Glycoside bond connection mode

**FIGURE 3:**
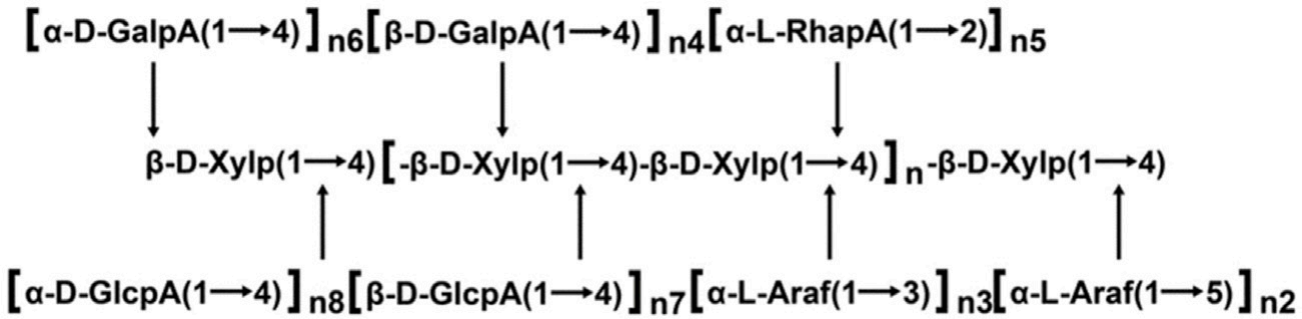
Possible structural model of *salvia miltiorrhiza* polysaccharide (Zhao et al., 2020).

The glycosidic linkage of polysaccharides can affect its form in solution, which is one of the important factors affecting its biological activity and one of the important indicators to characterize the biological activity of polysaccharides. At present, the research on the structure of *Salvia miltiorrhiza* polysaccharide mainly focuses on its primary structure. The connection methods for determining glycosidic bonds can be divided into chemical analysis method and instrumental analysis method. Chemical analysis methods include periodate oxidation method (Zhang et al., 2021), Smith degradation method (Perepelov et al., 2018), methylation method (Li et al., 2016), etc. Methylation involves methylating the glycoside chain, followed by GC-MS analysis after hydrolysis. Instrumental analysis methods include nuclear magnetic resonance (NMR) (Uhliarikova et al., 2021), GC-MS (He et al., 2017), Fourier transform infrared spectroscopy (FT-IR) (Chen et al., 2019a), etc. Zhao et al. extracted *Salvia miltiorrhiza* polysaccharide from 8% sodium hydroxide solution (H-8) and characterized it by GPC, FT-IR and NMR spectra. The results showed that the main chain was 4- β- D-Xylp, branch is 3- α- L-Arafat or 5- α- L-Araf-1, 4- β- D-Galp and β- D - Glcp, and α- L-Rhap, α- D-Galpa and α- Connected by D-GlcpA fragments (Zhao et al., 2020). Jing et al. characterized the structure of the extracted PSMP-2 by HPGPC, HPLC, FT-IR and methylation analysis, and found that the extracted PSMP-2 contains five main glycosidic bonds, (1 → 2,4) - linked Rha, (1 → 6) - linked Gal, (1 →) - linked Ara, (1 → 3,6) - linked Gal, (1 → 4) - linked Gal (Jing et al., 2022). See Table 3 for details. The possible structural model of *Salvia miltiorrhiza* polysaccharide is shown in Figure 3.

**TABLE 3:** Types and detection methods of danshen polysaccharide glycosidic bond.

Fraction name	Main monosaccharide composition	Nature	Line type	Detection method
SMP 1 (Wang et al., 2006)	Glu:Gal = 5.6∶1		(1→6)-α-D-Glc (1→2)-α-D-Glc	13C-NMR
SMP 0.5 (Wang et al., 2006)	Glu:Gal:Ara = 17∶1∶1		(1→6)-α-D-Glc	13C-NMR
SMP 1 (Geng et al., 2015a)			(1→3,6)-β-D-Manp (1→6)-β-D-Glcp (1→3,6)-β-D-Galp	GC-MS
HBPs (Zhao et al., 2020)	Xyl:Glu:Gal:Ara = 31.7:15.5:0.5:2		4-β-D-Xylp; 3-α-L-Araf or 5-α-L-Araf-1, 4-β-D-Galp and β-D- Glcp, α-L-Rhap, α-D-GalpA and α-D-GlcpA	FT-IR、NMR
PSMP-2 (Jing et al., 2022)	Rha:GalA:Gal:Ara = 6.15:55.98:21.27:16.69	Acidic	(1→)-linked-Ara, (1→2, 4)-linked-Rha, (1→4)-linked-Gal, (1→6)-linked-Gal, (1→3, 6)-linked-Gal	

### 4.3 Molecular weight

The molecular weight of *Salvia miltiorrhiza* polysaccharides has been primarily determined using gel permeation chromatography (GPC) and high-performance gel permeation chromatography (HPGPC) (Jiang et al., 2016). Jiang et al. extracted *Salvia miltiorrhiza* polysaccharide by hot water extraction, and separated and purified *Salvia miltiorrhiza* polysaccharide with DEAE-Sepharose CL-6B column and Sephadex G-100 column, and obtained a new polysaccharide with antioxidant activity, namely, SMWP-1, which was obtained by GPC. The molecular weight determined by the method is 5.27 × 10^5^ Da (Jiang et al., 2015). Ji et al. extracted *Salvia miltiorrhiza* polysaccharide by ultrasonic extraction, and then purified the polysaccharide by water dialysis to obtain SMGP. The average molecular weight of SMGP detected by HPGPC was 1.55 × 10^5^ Da (Ji et al., 2022). The molecular weight, monosaccharide composition, molar ratio and sugar chain structure of *Salvia miltiorrhiza* polysaccharides were obtained by different extraction and purification processes, as shown in Table 2. Therefore, we cannot uniformly define the structural characteristics of *Salvia miltiorrhiza* polysaccharides.

## 5 Pharmacological activity of *Salvia miltiorrhiza* polysaccharide

### 5.1 Protect myocardial cells

**FIGURE 4:**
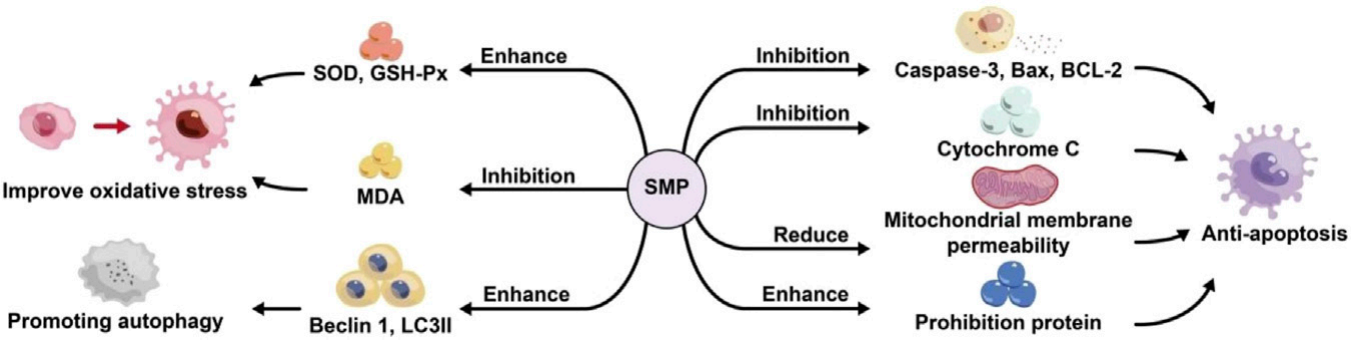
Protective mechanism of *salvia miltiorrhiza* polysaccharide on myocardial cells.

Coronary artery occlusion and myocardial injury or death can result in myocardial infarction (MI) or even heart failure, with a high mortality. Myocardial regeneration potential is extremely limited. Myocardial cells undergoing necrosis or apoptosis during myocardial infarction (He and Chen, 2020). Reperfusion is considered as the first effective strategy to save ischemic myocardium, but is accompanied by a series of adverse effects (Singhanat et al., 2021). Therefore, the demand for potential natural products with lower toxicity and fewer side effects is increasing. Numerous studies have demonstrated that polysaccharides have protective effects on myocardial cells, such as *Ganoderma lucidum* (Kahveci et al., 2021), *Chuanmingshen* (He et al., 2022). Zhou et al. used 200 μmoL/L H_2_O_2_ to induce neonatal rat cardiomyocytes *in vitro* to establish a myocardial injury model, and gave *Salvia miltiorrhiza* polysaccharide intervention. It was found that *Salvia miltiorrhiza* polysaccharide in different dosage groups (Low dose group: 1 × 10^−5^ moL/L; Medium dose group: 5 × 10^−5^ moL/L; High dose group: 1 × 10^−4^ moL/L) could significantly increase the expression of prohibitin protein in myocardial cells, especially in the high-dose group, suggesting its potential *Salvia miltiorrhiza* polysaccharide could protect myocardial cells from H_2_O_2_ induced damage (Zhou et al., 2011). Geng et al. isolated SMP1 from the root of *Salvia miltiorrhiza* and induced H9c2 myocardial cell damage through H_2_O_2_. It was found that pretreatment with SMP1 (25, 50 and 100 μg/mL) significantly prevented the mitochondrial damage, cytochrome c release, the increase of the ratio between apoptosis promoting Bax and anti-apoptosis BCl-2 protein expression, and the activation of caspase-3 in H9c2 cells stimulated by H_2_O_2_. SMP1 protects H9c2 cells from H_2_O_2_ induced apoptosis by inhibiting mitochondrial dysfunction, inactivating caspase-3 cascade and enhancing antioxidant capacity to protect cardiomyocytes (Geng et al., 2015a). Song et al. studied the protective effect of water-soluble *Salvia miltiorrhiza* polysaccharide SMP1 on the heart of rats with ischemia reperfusion (I/R) model. The results showed that SMP1 (400 and 800 mg/kg) could prevent I/R induced myocardial infarction by improving oxidative stress and inhibiting cardiomyocyte apoptosis (Song et al., 2013). It can be seen that *Salvia miltiorrhiza* polysaccharide can protect myocardium, and its mechanism may be related to improving oxidative stress of myocardial cells, inhibiting apoptosis of myocardial cells and promoting autophagy (Figure 4).

### 5.2 Protect the liver

**FIGURE 5:**
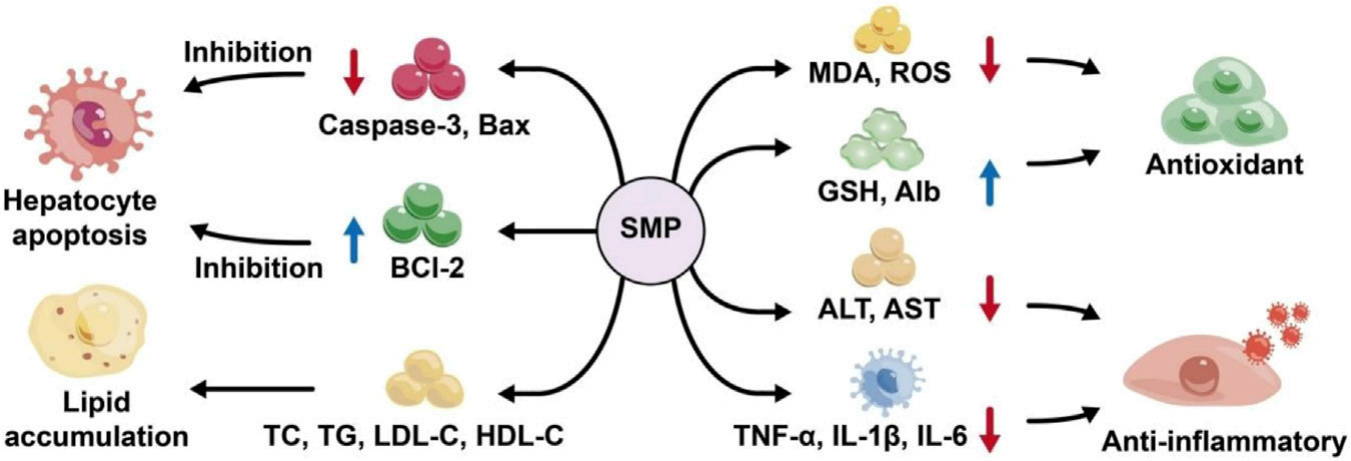
Protective mechanism of *Salvia miltiorrhiza* polysaccharide on liver.

Autoimmune attacks on hepatocytes, viral infections, drug abuse, and other factors can lead to liver injury (Malhi and Gores, 2008). Liver injury is influenced by multiple factors, and the underlying mechanisms of its pathogenesis and progression remain incompletely understood. A large number of studies have shown that polysaccharides can prevent and improve chemical liver injury and immune liver injury, such as Angelica sinensis (Cao et al., 2018), Schisandra chinensis (Yuan et al., 2018), etc. Song et al. established an immune liver injury model in mice through Bacille Calmette Guerin and lipopolysaccharide. The study found that *Salvia miltiorrhiza* polysaccharide (SMPS) (Low dose: 90 mg/kg; Medium dose: 180 mg/kg; High dose: 360 mg/kg) can effectively improve the thymus index, spleen index and liver index, reduced serum levels of nitric oxide (NO), aspartate aminotransferase (AST), and alanine aminotransferase (ALT), and restored tumor necrosis factor-alpha (TNF-α) and interleukin-1 beta (IL-1β) in the liver and has protective effect on immune liver injury (Song et al., 2008). Han et al. established a liver cell injury model by carbon tetrachloride, and studied the effects of *Salvia miltiorrhiza* polysaccharides (SMPs) (Dosage: 0.5, 1, 2 g/L) on chicken liver cell injury *in vitro* and *in vivo*. The results showed that the contents of TP, Alb and GSH were significantly increased, while the levels of liver index, ALT, AST and MDA were significantly decreased, indicating that SMPs had good protective effects on chicken liver injury *in vivo* and *in vitro* (Han et al., 2019). Yao et al. studied the effects of different concentrations of *Salvia miltiorrhiza* polysaccharide solution (High dose group: 15.6 g/kg, medium dose group: 7.8 g/kg and low dose group:3.9 g/kg) on acute liver injury in mice. The prepared *Salvia miltiorrhiza* polysaccharide was given to the mouse model of acute liver injury induced by tail vein injection of lipopolysaccharide (LPS), and its protective effect on the liver was observed. The results showed that *Salvia miltiorrhiza* polysaccharide could reduce the content of MDA in liver tissue, increase the content of GSH, and reduce the content of ALT in serum of mice with acute liver injury induced by LPS (Yao et al., 2010). It can be seen that the protective mechanism of *Salvia miltiorrhiza* polysaccharide on the liver may be related to its inhibition of the activation of TLR4/MyD88 signal pathway, inhibition of excessive peroxidation in the liver, and reduction of the production of inflammatory factors (Figure 5).

### 5.3 Immunomodulatory effect

**FIGURE 6:**
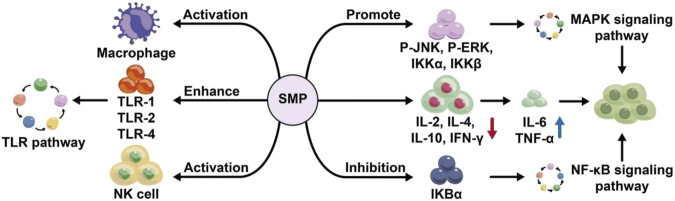
Immune regulation mechanism of *salvia miltiorrhiza* polysaccharide.

The regulation of immune response plays a critical role in the prevention of diseases. Recent studies have highlighted that the immune regulation and immune stimulation induced by bioactive compounds are increasingly valued (Yu et al., 2016). Immunomodulators primarily consist of protein adjuvant (Cui et al., 2018), aluminum hydroxide (He et al., 2015), and Freund’s adjuvant (FA) (Behr and Divangahi, 2015). However, neither aluminum hydroxide nor FA can induce strong cellular immunity. Protein adjuvants are too expensive to be commercialized. Therefore, it is urgent to develop a new immune adjuvant with high efficiency, low toxicity and abundant resources (Fan et al., 2010). Plant polysaccharides have obvious advantages in improving humoral and cellular immunity, such as mulberry leaves (Chen et al., 2019b), atractylodes macrocephala (Sun et al., 2015), etc. Plant polysaccharides can regulate the immune system by stimulating immune cells, regulating the release of cytokines, promoting of antibody secretion, etc. It plays an obvious role in improving humoral immunity, cellular immunity and mucosal immunity, and hold promise as potential metabolites for developing immune modulators (Jiang et al., 2010). *Salvia miltiorrhiza* polysaccharides also have immunomodulatory effects. Chen et al. found that SMP can significantly promote the proliferation of lymphocytes, and can enhance the cytotoxicity of T lymphocytes to cancer cells. Increase the gene expression of cytokines (such as IL-4, IL-6 and IFN-γ), enhance the gene expression of TLR1, TLR2 and TLR4; increase the protein expression of p-JNK, p-ERK, IKKα and IKKβ; reduce the level of IκBα. This indicates that SMP has specific regulatory effect on T lymphocytes through MAPK and NF-κB signaling pathways (Chen et al., 2017). Wang et al. found that SMPA (200 mg/kg) could improve immune organ indexes in gastric cancer rats. SMPA significantly stimulates splenocyte proliferation, promotes the production of anti-inflammatory cytokines such as IL-2, IL-4 and IL-10, inhibits the secretion of pro-inflammatory cytokines such as IL-6 and TNF-α, and enhances NK cells and T lymphocytes cytotoxicity and increased phagocytosis of gastric cancer rat macrophages. In addition, SMPA significantly increased total intracellular granzyme-B and IFN-γ levels produced by splenocytes. SMPA may act as a potent immunomodulator and may be a potential complementary source for gastric cancer treatment (Wang et al., 2014). Zhang et al. found that *Salvia miltiorrhiza* polysaccharide (200 mg/kg) can significantly promote lymphocyte proliferation reaction in mice; enhance the phagocytosis of peritoneal macrophages in mice; Inhibits ear swelling and decreases vascular permeability caused by dinitrofluorobenzene-induced allergic contact dermatitis of the pinna in mice, and significantly inhibits the expression of iNOS, IFN-α and IL-1β mRNA and other genes, mainly affects the organ index of the immune organs thymus and spleen, and has the effect of protecting the body from damage caused by the overexpression of cytokines, demonstrating good immune regulatory activity (Zhang et al., 2012). It can be seen that *Salvia miltiorrhiza* polysaccharide has immunomodulatory activity, and its mechanism of action may be related to the promotion of T lymphocytes and macrophages (Figure 6).

### 5.4 Antitumor effect

Cancer is one of the diseases that seriously harm human health, and with poor clinical outcomes. Its cancer cells are easy to spread within the body, the pathogenesis is diverse, prolonged treatment process, difficulty in cure, and high mortality. The main mechanism of standard biomedical treatment in treating cancer is to use drug toxicity to kill cancer cells. At present, no current drug selectively targets cancer cells without affecting normal cells (Alas et al., 2021). Plant polysaccharides can activate the immune system and play an immune regulatory role, and they can inhibit the proliferation of tumor cells, but have almost no toxic and side effects on normal cells (Yu et al., 2018), such as Astragalus polysaccharide (Shi et al., 2024), Ginseng polysaccharide (Zheng et al., 2025). Liu et al. obtained *Salvia miltiorrhiza* polysaccharide SMP-W1 through extraction and purification. After incubating H22 cells with SMP-W1 of different mass concentrations for 48 h, the cell activity was detected by MTT colorimetry. The results showed that with the increase of SMP-W1 concentration to 400 μg/mL, the cell activity decreased significantly, suggesting that SMP-W1 showed an inhibitory effect on the proliferation of H22 cells; It was found *in vitro* that it increased the activities of rat serum superoxide dismutase (Takahashi et al., 2023), catalase (CAT), glutathione peroxidase (GSH-Px), as well as the secretion of TNF-α. It has good anti-tumor activity *in vitro* (Liu et al., 2013). Jiang et al. purified the extracted *Salvia miltiorrhiza* polysaccharide to obtain SMP-U1. The study found that SMP-U1 directly inhibited the proliferation of Bcap-37 and Eca-109 cells, had a good anti-tumor activity, and the activity was good at the concentration of 0.30 mg/mL (Jiang et al., 2014). Wang et al. investigated the anti-tumor effects of 200 μg/mL SMP on human colorectal cancer LoVo cells, and finding that SMP exhibited a high inhibition rate on LoVo cells in a dose- and time-dependent manner; The polysaccharide can induce apoptosis of LoVo cells, block cell cycle in S phase, and increase intracellular reactive oxygen pressure. It is speculated that *Salvia miltiorrhiza* polysaccharide may have the potential to develop into a natural anti-cancer drug (Wang et al., 2018a). It can be seen that *Salvia miltiorrhiza* polysaccharide can play an anti-tumor role by improving the immune capacity of the body, inhibiting the growth of tumor cells and anti-oxidation.

### 5.5 Antioxidant

**FIGURE 7:**
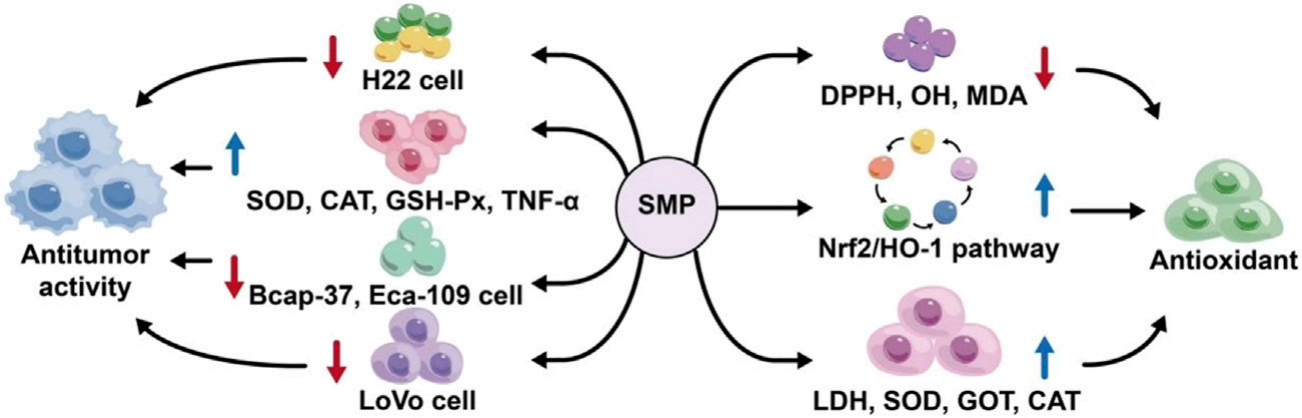
Antitumor and anti-oxidation mechanism of *Salvia miltiorrhiza* polysaccharide.

The human body produces free radicals in the body due to continuous contact with the outside world, and excessive free radicals will lead to aging, cancer or other diseases (Liu et al., 2018). Liu et al. conducted the first study on the antioxidant activity of Salvia miltiorrhiza bungeana polysaccharide (SMP) extracted via water extraction and ethanol precipitation, and studied the antioxidant activity of the polysaccharide of *Salvia miltiorrhiza* bungeana for the first time. The results indicated that SMP effectively inhibited linoleic acid peroxidation, and exhibited significant reducing power. Its both the inhibition rate and reducing power increased with higher mass concentrations, demonstrating a clear dose-response relationship. The inhibitory rate of 1 mg/mL *Salvia miltiorrhiza* polysaccharide on linoleic acid peroxidation was 23.05%. The results showed that the polysaccharide of *Salvia miltiorrhiza* Bunge had antioxidant activity and obvious inhibition on the peroxidation of linoleic acid (Zhen-Liang et al., 2013). Yong et al. extracted and purified *Salvia miltiorrhiza* polysaccharide by DEAE-52 cellulose column and Sephadex G-100 column chromatography to obtain an acidic polysaccharide PSMP-2, The study found that PSMP-2 demonstrated excellent scavenging capacity for DPPH and hydroxyl radicals, enhanced the activity of antioxidant enzymes *in vivo*, and has good antioxidant activity (Jing et al., 2022). Jiang et al. separated a new polysaccharide SMWP-1 with antioxidant activity from *Salvia miltiorrhiza* residue, which showed potent scavenging and reducing abilities against superoxide ions, DPPH, and hydroxyl radicals *in vitro*. SMP-1 has good effect at the concentration of 0.25 mg/mL (Jiang et al., 2015). Qi et al. optimized the extraction process of *Salvia miltiorrhiza* polysaccharide by response surface methodology, and then carried out antioxidant research. The results showed that it had obvious scavenging capacity for hydroxyl radicals, and the higher the concentration of polysaccharide, the stronger its scavenging capacity. The optimal effect was observed at a concentration of 4.5 mg/mL (Xiao-Ni et al., 2016). In conclusion, *Salvia miltiorrhiza* polysaccharide can play an antioxidant role by scavenging free radicals, improving antioxidant enzyme activity and other ways (Figure 7).

### 5.6 Others

*Salvia miltiorrhiza* polysaccharide not only has the activities of immune regulation, anti-tumor, anti-oxidation, myocardial cell protection, liver protection, but also has the effects of nerve protection (Zhang and Wei, 2016), blood pressure reduction (Xiao-Hong et al., 2017), kidney protection (Wang et al., 2022), anti-inflammatory (Han et al., 2018), etc. Shen et al. found that in the process of freezing boar semen, 0.4 mg/mL *Salvia miltiorrhiza* polysaccharide (SMP) can play a role in protecting boar sperm from oxidative damage, and can enhance sperm vitality, improve pregnancy rate. It is speculated that it is expected to be used in human or endangered wild animal sperm conservation (Shen et al., 2015). Han et al. studied the anti-inflammatory effect of SMP on 264.7 cells induced by lipopolysaccharide. The results showed that SMP significantly inhibited the mRNA transcription of TNF-α, IL-6, iNOS and COX-2 and the protein expression of NF-κB, p-p65 and p-IκBa, indicating that SMP has anti-inflammatory effects (Han et al., 2018). (Table 4) Salvia polysaccharides can also be used in combination with other drugs. Han et al. found that the combination of FFC and SMPs could improve the growth performance of broilers, increase the number of leukocyte subtypes in blood (P < 0.05), increase the number of Newcastle disease (ND) and avian influenza (AI) antibodies, the number of immunoglobulins and the contents of cytokines in blood (P < 0.05). The lymphocyte conversion rate in peripheral blood of broilers was significantly increased (P < 0.05), and the immune response of broilers was enhanced (Han et al., 2021). Wang et al. found that the combination of salviorrhiza polysaccharides with bifidobacterium bifidum V (BbV) and *Lactobacillus* plantarum X (LpX) in human microbiota decreased the mRNA concentrations of pro-inflammatory factors (tumor necrosis factor α, interleukin1β [IL-1β] and IL-6) (Wang et al., 2020).

**TABLE 4:** Biological activity and mechanism of *Salvia miltiorrhiza* polysaccharide.

Fraction name	Crude or purification	Activity	Mechanism
SMWP-U&E (Jiang et al., 2020)	Purification	Immunomodulation, antioxidant	Reduce MDA content and increase IgA, IgG, IgM, IL-2, IFN in serum- γ and IL-10 content
SMP (Chen et al., 2017)	Crude	Immunomodulation	Upregulation of gene expression of cytokines such as IL-4, IL-6 and IFN-γ, TLR1, TLR2 and TLR4; increased protein expression of p-JNK, p-ERK, IKKα and IKKβ and decreased IκBα level
SMP1 (Meng et al., 2022)	Purification	Antioxidant	Activation of the Nrf2/HO-1 pathway in PC12 cells
PSMP-2 (Jing et al., 2022)	Purification	Antioxidant	The scavenging capacity of IC50PSMP-2 for DPPH and hydroxyl radical is 0.991 mg/mL and 4.007 mg/mL respectively
SMP-U1 (Jiang et al., 2014)	Purification	Antineoplastic	Inhibiting the proliferation of Bcap-37 and Eca-109 cells
SMWP-1 (Jiang et al., 2015)	Purification	Antioxidant	It has strong reduced ability and free radical scavenging activity for DPPH radical, superoxide anion radical and hydroxyl radical
SMP (Wang et al., 2018a)	Purification	Antineoplastic	Induce LoVo cell apoptosis, block cell cycle in S phase, and increase intracellular reactive oxygen pressure
SMPs (Lu et al., 2022)	Crude	Reduce kidney damage	Regulate drug metabolism mediated by drug metabolizing enzymes; Increase GPX activity and T-AOC capacity and reduce LPO and ROS levels
SMPS (Song et al., 2008)	Crude	Immunomodulation	Improve liver index, spleen index and thymus index, reduce serum alanine aminotransferase, aspartate aminotransferase and nitric oxide levels, restore the content of tumor necrosis factor-α and interleukin-1β in liver homogenate
SMPs (Shen et al., 2015)	Crude	Antioxidant	Increase superoxide dismutase, lactate dehydrogenase, glutamate-oxaloacetate transaminase and catalase activities
DSP (Tu et al., 2013)	Crude	Antioxidant effect and protection of cerebral ischemia/reperfusion injury	Inhibit lipid peroxidation, enhance endogenous antioxidant defense and reduce ROS produced by mitochondria
LBM (Wang et al., 2020)	Crude	Treatment of NAFLD	Reduces mRNA concentrations of pro-inflammatory cytokines (tumor necrosis factor alpha, IL-1beta, and IL-6)
SMPs (Han et al., 2021)	Crude	Immunomodulatory	Improve the growth performance of broilers, increase the number of leukocyte subtypes in the blood, increase the number of Newcastle disease (ND) and avian influenza (AI) antibodies, the number of immunoglobulins and the content of cytokines in the blood
SMP-W1 (Liu et al., 2013)	Purification	Immune regulation, anti-tumor	Increase rat serum superoxide dismutase (Takahashi et al.), catalase (CAT) and glutathione peroxidase (GSH-Px) activities, and secretion of TNF-α
SMPs (Han et al., 2019)	Crude	Protective effect of chicken liver injury	The contents of TP, Alb and GSH were significantly increased, and the levels of liver index, ALT, AST and MDA were significantly decreased
SMP1 (Geng et al., 2015a)	Purification	Antioxidant	Inhibits mitochondrial dysfunction, inactivates the caspase-3 cascade
SMPs (Liu et al., 2022)	Crude	Relief of metabolic disorders in chick liver	Inhibits phase I and phase II metabolic function of the liver and FFC-induced hyperactivity of glycine and serine metabolic responses
SMPA (Wang et al., 2014)	Purification	Immunomodulatory	Promotes production of anti-inflammatory cytokines (IL-2, IL-4 and IL-10), inhibits secretion of pro-inflammatory cytokines (IL-6 and TNF-α), enhances natural killer cells and cytotoxic T lymphocytes (CTL) killing activity and increased phagocytosis of gastric cancer rat macrophages
SMP (Han et al., 2018)	Crude	Anti-inflammatory	Inhibits mRNA transcription of TNF-α, IL-6, iNOS and COX-2 and protein expression of NF-κB, p-p65 and p-IκBa
SMP1 (Geng et al., 2015b)	Purification	Protect myocardial damage	Reduce the levels of CK, CK-MB, LDH, and increase the concentrations of ALP, AST, ALT, TC, TG, LDL-C, and HDL-C; enhance the activity of SOD, CAT and GPX, improve the level of GSH, and reduce the concentration of TBARS
SMP1 (Song et al., 2013)	Purification	Beneficial effect of improving oxidative stress on myocardial ischemia reperfusion injury	SOD, Na (+) - K (+) - ATPase and Ca (2+) - Mg (2+) - ATPase activity and MDA level of I/R rats, creatine kinase and LDH serum activity increased
SMPW1 (Zhang et al., 2012)	Purification	Prevent insulin resistance by reducing oxidative stress	The expression or activity of CAT, SOD and glutathione increases, and the formation of GPx and MDA in serum and liver homogenate decreases
SMPs (Wang et al., 2019)	Crude	Antiinflammatory	Decreased mRNA levels of LBP, CD14, MD-2, TLR4 and MyD88; protein levels of TLR4, MyD88, P-IKK-α/β, P-IκB-α and P-P65; CXCL-10 and ICAM-1 levels; TNF-α and IL-1β concentrations
SMPs (Wang et al., 2022)	Crude	Inhibits oxidative stress and apoptosis	It reduces the content of uric acid, blood urea nitrogen and creatinine in serum and malondialdehyde in renal tissue, increases the level of glutathione, superoxide dismutase and catalase in renal tissue, decreases the relative expression level of p53, Caspase-3 and Caspase-6 mRNA and protein, and decreases the apoptosis rate of renal tissue cells
SMPs (Zhang et al., 2022)	Crude	Anti-inflammatory	Inhibits the protein expression of IL-1β, IL-6 and TNF-α, inhibits the increase of NF-κB and MAPK protein phosphorylation
SMPs (Geng et al., 2022)	Crude	Alleviate liver injury of broilers induced by florfenicol	Serum ALT, AST, liver LPO, ROS, IL-6 levels were significantly decreased, T-AOC, GSH-PX, IL-4 levels were significantly decreased

## 6 Toxicity

SMP is exhibits essentially non-toxic properties non-toxic. The research on acute and subacute toxicity test of SMP is reveal a scarcity of reports in domestic and foreign literatures. According to the acute toxicity test of *Salvia miltiorrhiza* polysaccharide in mice, all the mice survived after 7 days of pre-test, and there was no obvious change in the behavior or physical characteristics. The maximum tolerated dose of *Salvia miltiorrhiza* polysaccharide in mice was 15 g/kg. The maximum daily oral dose of SMP in adults is 9 g, and the maximum tolerance dose of mice is 200 times of the former. Therefore, it can be considered that SMP is safe within the common dose range. In the subacute toxicity test on rats, the rats were generally in good condition after continuous intragastric administration for 14 days, and there were no abnormalities in behavior, hair, diet, stool, secretion and excreta. There were no statistically significant differences between the rats of each dose group and the control group in terms of food intake, food utilization rate, body weight, blood biochemical indexes, main organ coefficient and pathological examination (P > 0.05), further indicating that *Salvia miltiorrhiza* polysaccharide is safe and non-toxic, and the test results can provide reference for further pharmacological experiments (Li, 2016).

## 7 SMP product development

SM is one of the commonly used herbs, which is widely used to treat diabetes, cardiovascular disease, etc. It is safe, effective, non-toxic and has an important role in medical care (Yin et al., 2021). There are 896 kinds of SM single and metabolites preparations with SM as the main metabolites the National Medical Products Administration has approved, including SM tablets, SM granules, SM injection, SM capsules, SM drop pills, SM cream, SM oral liquid, etc. The majority of the drugs are primarily utilized to promote blood circulation and resolve stasis, regulate qi and relieve pain, and mainly used to treat coronary heart disease, angina pectoris, chest pain, chest tightness and other symptoms. The State Administration for Market Regulation has approved 189 types of health supplements with SM as the main metabolites, which are mainly used to assist in reducing blood fat, increasing bone density, enhancing immunity, and assisting in the treatment of liver injury. The main suitable people are those with high blood fat, those at risk of chemical liver injury, middle-aged and elderly people, etc. However, these products are formulated using SM rather than SMP, and further development is required for SMP-related products. A total of five patents related to SMP were searched in the China National Intellectual Property Administration, and the application scope included the application in the preparation of weight-loss drugs, anti-inflammatory drugs, antioxidant drugs or healthcare products. Research shows that SMP, as one of the main components of SM, has unique pharmacological activity and great potential for product development, but drugs, healthcare products and patent applications with SMP as the main component are at an early stage. Therefore, it is of great significance and broad development space to actively research and develop drugs and health food with SMP as the main metabolites.

## 8 Clinical efficacy of SMP

Literature searches reveal a paucity of studies on the clinical efficacy of SMP. Only one study on the clinical efficacy of SMP was found through PubMed, and no relevant Chinese literature was found on CNKI. Chen et al. collected lymphocytes from cancer patients and studied the effect of SMP on T lymphocyte proliferation by cell counting and flow cytometry to explore the immune-regulatory properties of SMP. The results showed that SMP was dose-dependent on the proliferation of T lymphocytes in cancer patients, and significantly increased the cytotoxicity of T lymphocytes to cancer cells. However, SMP has no effect on the proliferation of tumor cells from the same source. The gene expressions of IL-4, IL-6, IFN-γ, TLR1, TLR2 and TLR4 were upregulated. The protein expressions of p-JNK, p-ERK, IKKα and IKKβ were increased. The specific regulatory effect of SMP on T lymphocytes was confirmed through MAPK and NF-κB signaling pathways (Chen et al., 2017). SMP has many biological activities, so it is necessary to study the clinical effect of SMP, which has important significance and broad development space.

## 9 In conclusion

Salvia is a traditional bulk Chinese herbal medicine with a long history of application. Early research on *Salvia miltiorrhiza* primarily focused on the chemical components of fat soluble phenanthraquinones and water-soluble phenolic acids, and less on the polysaccharide components of *Salvia miltiorrhiza*, this paper systematically reviews the extraction methods, purification and separation techniques, structural identification, and pharmacological activities of *Salvia miltiorrhiza* polysaccharides both domestically and internationally. At present, the extraction methods of *Salvia miltiorrhiza* polysaccharide include water extraction and ethanol precipitation, ultrasonic-assisted extraction, microwave-assisted extraction, and enzyme-assisted extraction. It is primarily composed of glucose, galactose, arabinose, and other monosaccharides. The glycosidic bond is mainly (1 → 6) - D-Glcp. It has the pharmacological effects of immune regulation, anti-tumor, anti-oxidation, myocardial cell protection, liver protection, etc. At present, there are still some deficiencies in the research of *Salvia miltiorrhiza* polysaccharides: first, the wild resources of Salvia miltiorrhiza have decreased, the planting area is increasing, and too much attention is paid to the output and the quality and medicinal properties of the medicinal materials are ignored, which leads to the increasingly serious quality problem of *Salvia miltiorrhiza*, but there is less research on the quality and identification of *Salvia miltiorrhiza*. Therefore, the establishment of a systematic fingerprint of *Salvia miltiorrhiza* polysaccharide can comprehensively reflect the types and quantities of sugar components in *Salvia miltiorrhiza* and its preparations, and then evaluate the quality of *Salvia miltiorrhiza* as a whole. The second is the extraction method of *Salvia miltiorrhiza* polysaccharide. Ultrasonic method is the most commonly used method to extract *Salvia miltiorrhiza* polysaccharide at present, but only a single method is used to extract *Salvia miltiorrhiza* polysaccharide, which cannot meet the needs of industrial production. Therefore, it is necessary to further study and improve the extraction methods, use different extraction methods to extract *Salvia miltiorrhiza* polysaccharide, improve the extraction rate of *Salvia Miltiorrhiza* polysaccharide, and explore the process suitable for industrial production; Finally, the biological activity mechanisms and safety of SMP remain unclear. At present, the research on pharmacological activity of *Salvia miltiorrhiza* polysaccharide is still at the stage of animal experiment and cell experiment. The research on its chemical composition, mechanism of action and clinical research is not deep enough, which limits its application scope. Therefore, further studies on the absorption, degradation mechanism, safety and toxicological evaluation of *Salvia miltiorrhiza* polysaccharides in human body are needed in future studies to clarify the mechanism of action of *Salvia miltiorrhiza* polysaccharides, so as to provide scientific basis for clinical research, development and utilization of *Salvia miltiorrhiza* polysaccharides.
